# Influence of cigarette smoking on the Index of Cardiac Electrophysiological Balance in apparently healthy Angolans

**DOI:** 10.21542/gcsp.2024.5

**Published:** 2024-01-03

**Authors:** Mauer A.A. Gonçalves, João Mário Pedro, Carina Silva, Pedro Magalhães, Miguel Brito

**Affiliations:** 1 Centro de Estudos Avançados em Educação e Formação Médica (CEDUMED), Faculty of Medicine, Agostinho Neto University, Luanda, Angola; 2 Department of Physiological Sciences, Faculty of Medicine, Agostinho Neto University, Luanda, Angola; 3 Centro de Investigação em Saúde de Angola (CISA), Angola; 4 Instituto Gulbenkian de Ciência, Oeiras, Portugal; 5 Health and Technology Research Center (H&TRC), Escola Superior de Tecnologia da Saúde de Lisboa, Instituto Politécnico de Lisboa, Portugal; 6 CEAUL - Centro de Estatística e Aplicações, Faculdade de Ciências, Universidade de Lisboa, Portugal

## Abstract

**Background:** Tobacco use accelerates atherosclerosis and is one of the predictors of death from ischemic heart disease, arrhythmias, heart failure, and sudden death. A new non-invasive parameter, the Index of Cardiac Electrophysiological Balance (iCEB) between depolarization and repolarization of the action potential, was considered a new biomarker for the identification of patients at increased arrhythmic risk.

**Objectives:** We aimed to evaluate the iCEB in apparently healthy Angolans with habitual cigarette smoking compared to non-smokers.

**Subjects and methods:** Data were obtained from the CardioBengo study, a cross-sectional community-based study in which a random sample of individuals aged between 15 and 84 years was selected. In total, 214 apparently healthy subjects, 102 smokers, and 112 non-smokers in the same age group were included in the final analysis.

**Results:** The average age of the participants was 42.17 ± 13.04 years old and 26.6% of the sample was female. Smoking subjects had higher iCEB and corrected Index of Cardiac Electrophysiological Balance (iCEBc) values compared with non-smoking controls (4.39 *vs.* 4.25; *p* = 0.024, respectively), and (4.74 *vs*. 4.57; *p* = 0.030, respectively).

**Conclusions:** In summary, iCEB and iCEBc were significantly higher in habitual smokers than in nonsmokers, which represents an increased risk of ventricular arrhythmogenesis in healthy habitual smokers. To the best of our knowledge, this is the first study performed in Africa to evaluate iCEB in smokers, making this type of study very important in low- and middle-income countries in the context of epidemiological transition.

## Introduction

Tobacco use is a critical public health issue and a major risk factor for life-limiting diseases that result in early death^1^. Studies on coronary artery disease (CAD) risk factors have shown that smoking accelerates the process of atherosclerosis and is a predictor of death from ischemic heart disease, arrhythmias, heart failure and sudden death^2–5^.

Despite the increase in knowledge and awareness of the harm attributed to tobacco consumption, worldwide, tobacco is responsible for 7 million deaths per year, and it is estimated that this number will reach 8 million by 2030^6^.

Cigarette smoke inhalation enhances myocardial oxygen demand owing to carbon monoxide and adrenergic stimulation, resulting in changes in blood pressure and coronary artery tone, which can cause angina and cardiac arrhythmia^2^. Nicotine, an important constituent of cigarette smoke, delays ventricular repolarization, releases catecholamines into circulation, activates the sympathetic nervous system, and prolongs membrane repolarization by blocking K ^+^ entry channels in the ventricular myocardium^7^.

For a long time, electrocardiographic abnormalities in QT interval duration have been associated with an increased risk of ventricular arrhythmias and sudden cardiac death, and previous studies have shown conflicting results regarding the influence of acute and chronic smoking on QT interval duration^8–10^. However, a new noninvasive parameter, the Index of Cardiac Electrophysiological Balance (iCEB) between depolarization and repolarization of the action potential, was considered a new biomarker for the identification of patients with increased arrhythmic risk^11–13^.

According to Lu et al. (2013), iCEB may have advantages over the current biomarkers in identifying the potential risk for cardiac arrhythmias, especially for its potential to discriminate between long-QT-related arrhythmias, including Torsades de Pointes (TdP) and non-TdP-like ventricular tachycardia/ventricular fibrillation^11^.

Cardiovascular disorders are becoming increasingly important in developing nations such as Angola. Thus, there is an urgent need to reduce risk factors through educational activities and health promotion programs that can, in this way, reduce the impact of these diseases on the population.

Knowledge of smoking as an indicator of the risk of arrhythmias can contribute to a better understanding of risk groups and the implementation of prevention, monitoring, and treatment strategies. The aim of this research was to evaluate the status of the Index of Cardiac Electrophysiological Balance (iCEB) in apparently healthy Angolans with habitual cigarette smoking compared to non-smokers.

## Subjects and methods

The present report is a subset analysis of CardioBengo, a community-based study with a cross-sectional design conducted between September 2013 and March 2014 in the municipality of Dande, Bengo^14^. The study selected a representative random sample of 2484 black individuals stratified by sex and age between 15 and 84 years, as previously described in the study protocol^14^. The prevalence of smoking in the CardioBengo study was 10.0% in men and 2.6% in women^15^.

For the analyses described here, we excluded participants with previous hypertension, diabetes mellitus, heart failure, history suggestive of congenital or valvular heart disease, stroke, thyrotoxicosis, sickle cell disease, pregnancy, and/or presence of major abnormalities in the ECG according to the Minnesota code (MC).

In this study, 152 smokers were identified and according to the exclusion criteria described above, 50 smokers were excluded from the sample. Therefore, 102 smokers and 112 non-smokers in the same age group selected from the sample were included in the non-smoker control group. In total, 214 apparently healthy subjects were included in the final analysis.

Information on age, sex, education, alcohol and tobacco consumption through a structured interview conducted by trained and certified interviewers following the standardized protocol of the World Health Organization (WHO), based on the Surveillance Manual (STEPS) for Risk Factors for Chronic Diseases (central and expanded version 3.0)^16^. Moreover, we measured blood pressure, glucose and cholesterol levels in addition to the collection of medical history and anthropometric data.

## Electrocardiography

Standard resting 12-lead electrocardiograms and a rhythm strip were recorded for all participants using a 12-channel AsCARD Mr. Grey V 201 electrocardiograph (ASPEL, Zabierzów, Poland). The exam was digitally recorded using cardio TEKA v001 database software (ASPEL, Zabierzów, Poland) and subsequently transferred to the Central Electrocardiography Laboratory at the University of Glasgow, where they were analyzed and processed by in-house software and coded by the Minnesota Code^17,18^.

In the present study, measurements of P-wave and QRS duration, PR and QT intervals, and P, R, and T axes were automatically performed. QT interval was corrected using the formula of Bazett^19^. The iCEB was calculated by dividing the QT interval by QRS duration (QT/QRS) and iCEBc by the ratio of QTc to QRS (QTc/QRS)^11,12^. The MC classified electrocardiograms as major, minor, or absence of abnormalities^18^. Abnormal ECGs due to the presence of alterations were manually reviewed by two cardiologists to guarantee the quality of the coding.

### Statistical analysis

Means and standard deviations were computed and presented as quantitative variables. Data were analyzed considering smokers and non-smokers, and some parameters were compared by sex.

Student’s *t*-test and non-parametric Mann–Whitney tests were used for comparison between groups, as appropriate. The significance level was set at *p* = 0.05 and all statistical ana lysis was carried out using IBM SPSS^®^ (Statistical Package for the Social Sciences) version 26.

### Statement of Ethics

The ethics committee of the Angolan Ministry of Health approved the CardioBengo study protocol and all use of secondary data, and the present study was approved by the independent ethics committee of the Faculty of Medicine of the Agostinho Neto University (DELIBERATION n^o^ 9/2020).

Written informed consent was obtained from all participants prior to data collection, following all human research standards according to the Helsinki Declaration on the ethical principles for medical research involving human subjects.

## Results

The baseline characteristics of 214 individuals (102 smokers and 112 non-smokers) stratified by sex are shown in Table 1. Females constituted approximately 26.6% of all participants.

**Table 1:** Baseline characteristics of smoker and non-smoker groups (mean ± DP).

**Variables**	Smoker group *n* = 102	Non-smoker group *n* = 112	p*	Total *n* = 214
Age (years)	42.37 ± 13.17	42.03 ± 13.02	0.847	42.17 ± 13.04
Body weight (kg)	55.59 ± 10.38	60.32 ± 10.77	**0.001**	58.03 ± 10.80
Height (m)	1.63 ± 0.08	1.63 ± 0.09	0.958	1.63 ± 0.08
BMI (Kg/m^2^ )	21.62 ± 8.39	24.09 ± 10.91	0.066	22.89 ± 9.82
Waist circumference (cm)	74.87 ± 8.13	78.33 ± 9.99	**0.007**	76.66 ± 9.28
Hip circumference (cm)	88.45 ± 7.35	92.01 ± 9.72	**0.003**	90.29 ± 8.82
WHR	0.84 ± 0.59	0.85 ± 0.12	0.500	0.85 ± 0.93
SBP (mmHg)	121.4 ± 14.4	123.8 ± 16.4	0.253	122.7 ± 15.5
DBP (mmHg)	76.5 ± 11.4	77.9 ± 11.8	0.359	77.1 ± 11.6
Glycemia (mg/dL)	112.84 ± 16.14	126.85 ± 19.29	0.241	120 ± 17.94
Total cholesterol (mg/dL)	173.55 ± 33.04	180.51 ± 29.79	0.163	177.30 ± 31.33

**Notes.**

HR Heart rate SBP systolic blood pressure DBP diastolic blood pressure WHR waist hip ratio

* *p*-values obtained by *t*-test and bold values are considered significative.

**Table 2:** ECG parameters in smokers and non-smokers (mean ±SD).

**Parameters**	Smoker group *n* = 102	Non-smoker group *n* = 112	p^*^
HR (bpm)	70.58 ± 11.29	70.98 ± 12.72	0.807
P wave duration (ms)	107.53 ± 11.97	108.21 ± 13.67	3l0.701
PRI duration (ms)	154.29 ± 20.03	154.75 ± 19.54	3l0.866
QRS duration (ms)	87.45 ± 8.20	90.32 ± 9.09	3l**0.017**
QTI duration (ms)	381.67 ± 28.24	380.09 ± 29.45	3l0.690
QTIc (ms)	410.42 ± 24.69	408.68 ± 22.88	3l0.593
iCEB	4.39 ± 0.44	4.25 ± 0.50	**0.024**
iCEBc	4.74 ± 0.54	4.57 ± 0.55	**0.030**

**Notes.**

HR Heart rate PRI PR interval QTI QT interval QTIc QT interval corrected iCEB index of cardiac electrophysiological balance iCEBc index of cardiac electrophysiological balance with heart rate correction

* *p*-values obtained by *t*-test and bold values are considered significative.

In the smoker group, however, 24.5% of the 102 individuals were female, with a mean age of 42.17 ±13.04 years. Smokers showed significantly reduced body weight and waist and hip circumference. There were no significant differences in other variables.

Smoking subjects had higher iCEB and iCEBc values compared with non-smokers (4.39 *vs.* 4.25; *p* = 0.024, respectively), and (4.74 *vs*. 4.57; *p* = 0.030, respectively), and had shorter QRS duration (87.45 *vs.* 90.32; *p* = 0.017. These differences are presented in detail in Table 2.

**Table 3:** ECG parameters between gender (mean ±SD) in the smoker group.

**Parameters**	Male smoker *n* = 77	Female smoker *n* = 25	p^*^
HR (bpm)	69.61 ± 11.24	73.56 ± 11.17	0.129^*^
P wave duration (ms)	108.27 ± 11.77	105.17 ± 12.54	0.144^**^
PRI duration (ms)	155.12 ± 20.06	151.76 ± 20.14	0.436^**^
QRS duration (ms)	89.45 ± 7.74	81.28 ± 6.40	<0.001*
QTI duration (ms)	378.62 ± 27.56	391.04 ± 28.80	0.056^*^
QTIc (ms)	404.18 ± 21.51	429.64 ± 24.32	<0.001^*^
iCEB	4.25 ± 0.37	4.83 ± 0.37	<0.001*
iCEBc	4.55 ± 0.42	5.31 ± 0.48	<0.001^*^

**Notes.**

HR Heart rate PRI PR interval QTI QT interval QTIc QT interval corrected iCEB index of cardiac electrophysiological balance iCEBc index of cardiac electrophysiological balance with heart rate correction

* *t*-test; **Mann-Whitney test. Bold values are considered significative.

In the group of smokers, the QRS duration was significantly higher in men (89.45 *vs*. 81.28; *p*<0.001), whereas the QTIc, iCEB, and iCEBc were significantly higher in women (404.18 *vs.* 429.64; *p*<0.001), (4.25 *vs*. 4.83; *p*<0.001) and (4.55 *vs*. 5.31; *p*<0.001), respectively (Table 3).

**Table 4:** Comparison of the ECG parameters (mean) between the Smoker group and Non-smoker group according to gender.

**Parameters**	Female		p^*^	*Male*		*p* ^**^
	Non-smokers (*n* = 32)	Smokers (*n* = 25)		*Non-Smokers* (*n* = 80)	*Smokers* (*n* = 77)	
HR (bpm)	72.41 ± 14.18	73.56 ± 11.17	0.740¤	70.41 ± 12.14	69.61 ± 12.24	0.668
P wave duration (ms)	109.38 ± 13.87	105.17 ± 12.54	0.153 ¥	107.75 ± 13.65	108.27 ± 11.77	0.798
PRI duration (ms)	156.31 ± 19.73	151.76 ± 20.14	0.240 ¥	154.13 ± 19.55	155.12 ± 20.06	0.754
QRS duration (ms)	89.44 ± 6.86	81.28 ± 6.40	**<0.001**¤	90.68 ± 9.86	89.45 ± 7.34	0.391
QTI duration (ms)	391.94 ± 32.25	391.04 ± 28.80	0.913¤	375.35 ± 27.04	378.62 ± 27.56	0.454
QTIc (ms)	423.44 ± 22.20	429.64 ± 24.32	0.320¤	402.78 ± 20.46	404.18 ± 21.51	0.675
iCEB	4.40 ± 0.47	4.83 ± 0.37	**0.001** **¤**	4.18 ± 0.50	4.25 ± 0.37	0.320
iCEBc	4.76 ± 0.46	5.31 ± 0.48	**<0.001** **¤**	4.50 ± 0.51	4.55 ± 0.42	0.521

**Notes.**

HR Heart rate PRI PR interval QTI QT interval QTIc QT interval corrected iCEB index of cardiac electrophysiological balance iCEBc index of cardiac electrophysiological balance with heart rate correction ¤ Teste T ¥ Mann–Whitney

p* - Female smokers vs Female non-smokers.

p** - Male smokers vs Male non-smokers.

We found that QRS duration was significantly higher in female non-smokers (81.28 *vs*. 89.44; *p*<0.001) (Table 4). The iCEB and iCEBc were significantly higher in female smokers than in non-smoking controls (4.83 *vs.* 4.40; *p* = 0.001) and (5.31 *vs.* 4.76; *p*<0.001), while in males, there were no statistically significant differences.

## Discussion

The current study allowed for the description of the iCEB in apparently healthy Angolan adults of both sexes by comparing habitual smokers *versus* non-smokers. To the best of our knowledge, this is the first study to be conducted in Africa.

In the present study, iCEB and iCEBc levels were significantly higher in smokers than in non-smokers. In a study conducted in Turkey on the effect of habitual cigarette smoking on the index of cardiac electrophysiological balance in apparently healthy individuals, the ICEB was higher in smokers, but without a statistically significant difference, whereas the iCEBc was significantly higher in smokers^13^. This increase in iCEB and iCEBc levels in habitual smokers may represent an increased risk of ventricular arrhythmogenesis in healthy habitual smokers, as observed in some studies^11,13^.

iCEB, defined as QT/QRS, is not a rate-independent factor. In healthy young individuals, QRS shortening is often observed at high heart rate^20^. This partially eliminates the effect of heart rate changes on iCEB. This is evidenced by the widening of the QRS duration in a rate-dependent manner^9,11^.

In our study, comparing smokers according to sex, the QRS duration was significantly higher in male smokers, which is in line with earlier studies^13^.

In contrast, smokers had a substantial decrease in the duration of the QRS complex compared to the control group. This change in QRS duration is in accordance with the results of Siddiqui et al. (2013), who compared ECG parameters among smokers, chewers and non-tobacco users^21^. While several studies have not shown significant differences in QRS duration in smoking and non-smoking subjects^13,22,23^. In smokers who have developed pulmonary emphysema, a decrease in ECG amplitude as well as a decrease in the duration of the P wave, QRS complex, and T wave may be observed because of marked disparities in the boundaries of the layers of the passive volume conductor, leading to attenuation of all components of the ECG curve, an extracardiac phenomenon. However, this reduction in QRS duration in apparently healthy smokers is still not well understood^24–26^. Another issue that arises regarding QRS decrease due to extracardiac causes is that such alterations may have an influence on individuals with heart failure with indication for cardiac resynchronization therapy, since such a decision, most of the time, is taken based on the duration of the QRS^27^.

The QT interval represents the time required for depolarization and repolarization of the ventricular myocardium. In the present study, QTIc was significantly higher in female smokers than in male smokers. The QT interval was corrected by the respective heart rate according to the formula put forward by Bazett. It should be noted that the prolonged QT interval is considered as a marker of imbalanced distribution of sympathetic nervous system activity on the heart, indicating that the autonomic neural tone is an important determinant of QT interval duration and dispersion^28,29^.

In turn, iCEB and iCEBc levels were significantly higher in female smokers than in female non-smokers. In a study by Levent Özdemir and Erdoğan Sökmen (2020), iCEB showed no significant difference, while iCEBc showed results similar to ours^13^. The higher iCEB and iCEBc values found in women can be explained by the higher QT interval, probably related to the effect of sex hormones, and by the reduced QRS duration, associated with smaller cardiac dimensions in females^12^. Based on these findings, we suggest that closer ECG monitoring is warranted in female smokers using drugs to prolong QT.

Our study’s findings support previous research in indicating that smoking habitually can pose an additional risk of ventricular arrhythmias caused by TdP in individuals who are already taking medication to prolong the QT interval or have a genetic predisposition to long QT syndrome, which increases their susceptibility to TdP-mediated ventricular tachycardia and fibrillation. This increased risk is present even in healthy habitual smokers^11–13,30^.

## Limitations

It is important to highlight a limitation of this dataset, the fact that we had a relatively small sample in this study, and we did not consider the duration of smoking or the amount of nicotine consumed by the participants. However, this study has the following strengths: (1) it is a population-based study with a randomly selected representative sample; (2) ECG collection and recording were standardized and did not vary during the study, as they were performed by a single trained technician, and the examination was digitally recorded using reliable database software.

## Conclusions

This is the first study to evaluate the status of iCEB in habitual cigarette smokers compared to non-smokers in an apparently healthy autochthonous African population, making this a very important study in low- and middle-income countries in the context of epidemiological transition.

Our study shows that iCEB and iCEBc were significantly higher in habitual smokers than in non-smokers, which represents an increased potential risk of TdP-mediated ventricular arrhythmias in healthy habitual smokers, suggesting a possible increase in the susceptibility to TdP-mediated ventricular arrhythmias in habitual smokers using drugs to prolong QT and/or inherent carriers of long QT syndrome mutations.

## Author Contributions

Conceptualization: MAAG, JMP, MB, PM; Field work: JMP, MB; Data curation: JMP, MB; Formal analysis and statistics: CS, PM; First draft of the manuscript: MAAG; Manuscript review and editing: MAAG, CS, MB, PM; All authors approved the manuscript final version

## Conflict of Interest Statement

The authors have no conflicts of interest to declare.

## Funding Sources

The present study was supported by: Camões, Institute of Cooperation and Language, Portugal; Calouste Gulbenkian Foundation, Portugal; Government of Bengo Province, Angola; and the Angolan Ministry of Health.

## Acknowledgements

We thank all Dande - Health Demographic Surveillance System and Bengo General Hospital staff for their continuing support during fieldwork, namely Joana Paz and Ana Oliveira, who supervised the field work, Eduardo Saraiva for data entry supervision and database management, Edite Rosário for the training of field workers and assistance in data collection. Most importantly, the local administration, and all of the individuals who accepted to take part in the study. Moreover, we would like to thank Humberto Morais for suggestions made to the draft and Brian Devine and Peter Macfarlane for support in ECG analysis.
